# Big bladder stone accompanied by big posterior urethral stone: A management of rare case report

**DOI:** 10.1016/j.ijscr.2024.109853

**Published:** 2024-06-06

**Authors:** Soetojo Wirjopranoto, Yufi Aulia Azmi, Ronald Sugianto, Kevin Muliawan Soetanto

**Affiliations:** a Department of Urology, Faculty of Medicine, Universitas Airlangga, Dr. Soetomo General Academic Hospital, Surabaya, Indonesia; b Department of Health Sciences, University of Groningen, University Medical Center Groningen, Groningen, the Netherlands; c Prof. I.G.N.G. Ngoerah General Hospital, Denpasar, Bali, Indonesia; d Department of Immunology, Faculty of Medicine Siriraj Hospital, Mahidol University, Bangkok, Thailand

**Keywords:** Bladder stone, Urethral stone, Urinary retention, Morbidity

## Abstract

**Introduction and importance:**

Urinary tract stones are a common disease, but concurrent large-size stones in the bladder and urethra are rare. This phenomenon can lead to obstruction, infection, and other complications. We reported the management of a rare case of a giant bladder stone accompanied by a big posterior urethral stone.

**Case presentation:**

A 36-year-old man with a chief complaint of not being able to have spontaneous micturition, frequent expulsion of stones from the penis, and a history of hematuria. Bladder examination revealed a giant bladder stone of 1278 Hounsfield Unit (HU) with a size of 4.1 × 7.2 cm, and urethral examination revealed a stone of 1275 Hounsfield Unit (HU) with a length of 4.3 × 4.2 cm, without mass. This patient underwent vesicolithotomy and urethrotomy. The evaluation showed spontaneous micturition and dissolved hydronephrosis.

**Clinical discussion:**

Urinary tract stone management primarily involves endourology or open surgery. For smaller stones (<5–6 mm), medication is sufficient, as they often pass spontaneously. Larger stones may require interventions like vesicolithotomy or urethrotomy. Vesicolithotomy is preferred for complex or large bladder stones, while urethrotomy is performed if the stone location is palpable or seen on imaging. These procedures are practical options for general surgeons in first-level hospitals.

**Conclusion:**

Concurrent large bladder and urethral stones are uncommon. Endourology or open surgery is typically employed. Treatment selection should be personalized to individual patient assessment to mitigate potential complications effectively.

## Highlights

• Big bladder stone concurrent with big urethral stones in one patient is a rare case.
• Open surgery (Vesicolithotomy combined with urethrotomy) yields positive outcomes in a complex case of big bladder stone concurrent with big urethral stone.
• Multidisciplinary care optimizes treatment strategies for favorable outcomes.

## 1 Introduction

Urinary tract stones are a common disease that attacks various parts of the urinary system, such as the urethra and bladder. Bladder stones are solid stones that mainly occur within the bladder. Bladder stones are generally rare in Western countries, although they are common in developing countries due to nutritional factors [1]. Bladder stones account for only around 5 % of all urinary tract stones, yet they cause 14 % of hospital admissions and 8 % of urolithiasis-related fatalities in developing countries [2]. Five percent of cases of urinary tract stones are bladder stones. Due to early-life malnutrition, bladder stones are prevalent in Turkey, Iran, India, China, and Indonesia; however, when social conditions improve, the frequency of bladder stones declines. It is uncommon for bladder stones to weigh >100 g [3]. Bladder stones arise less frequently and typically weigh <100 g. A broad presentation spanning from asymptomatic to lower abdominal pain, dysuria, gross hematuria, or urine retention might render the diagnosis difficult [4]. Compared to women, men are more likely to be impacted. Women experience about 5 % of all bladder stones, typically linked to urine stasis or a foreign body (stitching, synthetic tape, or mesh) [5].

In a study conducted in a tertiary care facility in Eastern Nepal, 455 stones were recovered. Seven percent of the 455 stones were bladder stones, and 87 % were urethral stones [6]. A systematic review and meta-analysis found 46 studies conducted in 22 provinces across China. There was a combined prevalence of urolithiasis of 8.1 %, with urethral stones having a prevalence of 0.5 % (95 % CI 0.1–0.9 %) [7]. In 621 cases of urolithiasis in the North-Eastern city of India, stones in the urethra were the least common compared with other locations, with vesical stones and urethral stones present only in men [8]. A case study from the Southern Indian steel industry found a high prevalence of kidney/urethral stones among the high-heat exposed steelworkers [9]. Even though urethral stones are smaller than bladder stones, both cases can cause complications.

The etiology of bladder stones is primary, secondary, and stone migration. When no other urinary tract disease is present, primary or endemic bladder stones develop. These stones are typically observed in children who live in areas with low animal protein diets, frequent diarrhea, and dehydration. Other urinary tract problems such as bladder outlet obstruction, neurogenic bladder dysfunction, persistent bacteriuria, foreign items such as catheters, bladder diverticula, and bladder augmentation or diversion can also cause secondary bladder stones. Furthermore, certain bladder stones might move via the upper urinary tract [2].

The pathophysiology of urethral stones primarily affects men. Because they are too big for a typical urethral diameter, they begin in the bladder and cannot move [10]. Urinary tract infections, strictures, urethral diverticula, and urethral foreign bodies can all be linked to urethral stones. Timely diagnosis is crucial since urethral stones can be difficult and lead to acute urine retention or infection [11].

Stone cases with two locations and large are very rare. We reported the management of a rare case of a big bladder stone accompanied by a big posterior urethral stone. We present the following case using the SCARE guideline [12].

## 2 Case presentation

**Fig. 1:**
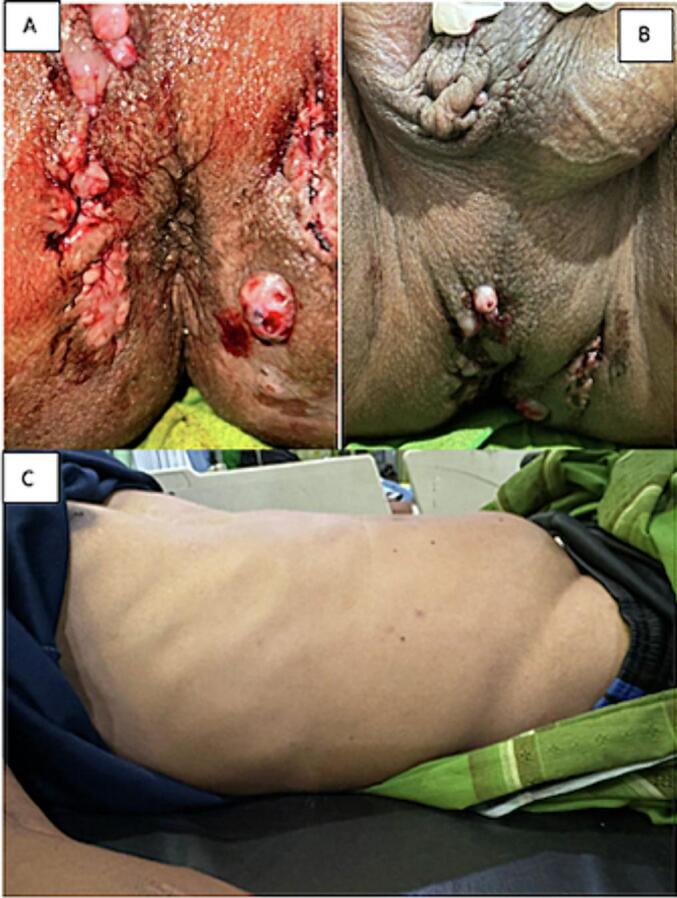
(a) Clinical Picture of Perianal Fistula; (B) Clinical picture of the patient's scar testicle; (C) Clinical picture of the patient's abdomen.

A 36-year-old man presented to the emergency room and was referred from First Line General Hospital in a rural area with a chief complaint of not being able to have spontaneous micturition for a day. The man had already tried catheter insertion but failed. Frequent expulsion of stones from the penis, most recently with hematuria one day ago. Since elementary school age, there have been complaints of pus coming out from around the perineum. It was intermittent, but it has never been treated until now. One year ago, pus also spread out from the scrotum, but now there is no pus coming out of the abscess area in the scrotum and around the anus, but there is still blood coming out that won't stop (Fig. 1a-b). History of hypertension and type 2 diabetes was denied. The patient married once and has four children; a history of surgery was denied.

On physical examination, the vital signs were unremarkable. On examination of the genitourinary flank mass and Costo Vertebral Angle (CVA), knock pain was found to be negative. The results of the suprapubic physical examination showed suprapubic bulging, hard consistency, and palpable stone (+). When percussion is tested, the stone makes a deaf sound (Fig. 1c). The results of the external genital were within normal limits, and the fibrotic scar from the right scrotal abscess was positive. The results of the RT examination showed the presence of a fibrotic scar from the perianal abscess and bleeding from the scar, TSA positive normal, and mucose smooth. In the digital rectal exam, manual palpation was performed. The results found a hard mass that could move, tenderness (+), and palpable stones at 12 o clock.

Laboratory test results showed the patient had anemia (hemoglobin = 9.7 g/dL). Platelets were also low (127,000 × 103/μL). This result occurs because there is intermittent hematuria, and the patient has a low intake. The patient also underwent urinalysis with pH 6.5, Erythrocytes 3+, and Leukocytes 3+. The patient had no other comorbidities.

**Fig. 2:**
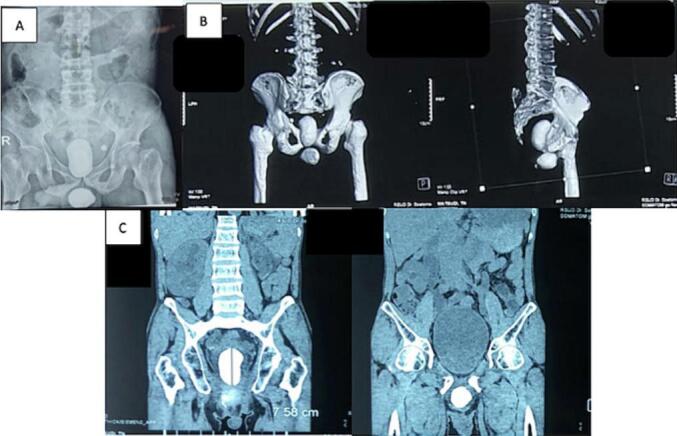
(a) KUB Photo; (b) 3D reconstruction of CT-Stonography; (C) Sagittal view of CT-Stonography.

The Kidney Ureter Bladder (KUB) photo showed a radiopaque shadow in the bladder and posterior urethra. CT Sonography on renal dextra showed the presence of severe Hydronephrosis (HN) and multiple stones with 589 Hounsfield Unit (HU) at the lower pole sized 0.58 × 1.8 cm and the presence of stones (545 HU) at the right *ureterovesical junction* (UVJ) sized 0.6 × 0.6 cm, without mass and cysts. An examination of renal sinistra showed the presence of severe HN, contracted renal sinistra, and stone (1619 HU) at left UVJ sized 1.7 × 1.3 cm, without mass and cysts. The results of the bladder examination showed the presence of a giant bladder stone (1278 HU) 4.1 × 7.2 cm, and the results of the urethra examination showed the presence of a stone (HU 1275) 4.3 × 4.2 cm, without mass (Fig. 2b-c). The operator did not perform a bladder biopsy because of the presence of large urethral stones, which can cause chronic inflammation and Squamous Cell Carcinoma (SCC) of the bladder. The family was educated about the risk factors of SCC bladder.

**Fig. 3:**
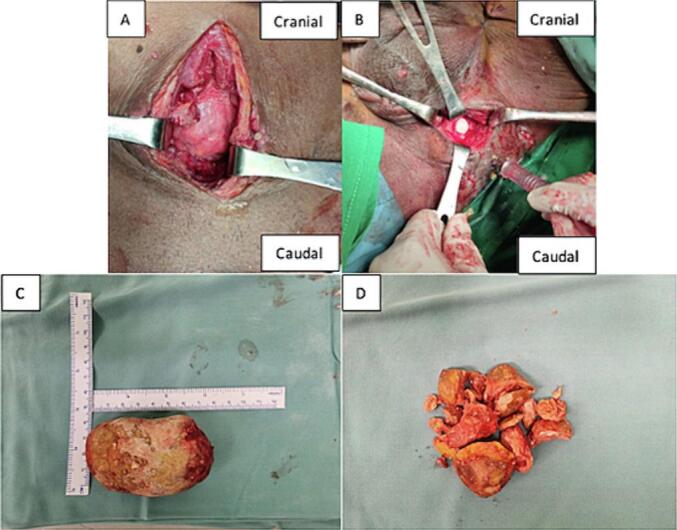
(A) Clinical picture of the patient's bladder; (B) Clinical picture of the patient's urethra and urethral stone; (C) Clinical picture of bladder stone; (D) Clinical picture of fragmented urethral stone.

In these patients, the resident doctor, under supervision from the supervisor, performs vesicolithotomy and urethrotomy. The patient is in the lithotomy position. During the vesicolithotomy, the patient underwent a suprapubic catheter (cystostomy) because the bladder stone was large. The stone was impacted into the bladder during the operation and freed as much as possible. In the first stage of vesicolithotomy, the patient undergoes a suprapubic incision and then bladder identification (Fig. 3a). A bladder incision found the bladder stone and intra-bladder blood clot; then evacuation was carried out. A total of +/− 500 cc of clot was obtained. A bladder stone of 8 × 4 cm was obtained (Fig. 3c). The next step, exploration was carried out, and a second stone measuring 2 × 1 cm was obtained; both stones came out intact. Next, we performed a urethrotomy. The steps taken are identifying urethral stones from the perineum, making an incision from the median raphae, and making an incision on the stone. The stone could not be removed from the urethra (Fig. 3b), so the stone was removed by osteotomy. The next step is that the stone is fragmented, then the stone is evacuated (Fig. 3d). When performing a urethrotomy, urethral tissue is removed so the urethra can be stitched back properly. A small amount of urethral tissue was taken because the stone was impacted. The affected urethral stones are subjected to trypsy until they are destroyed and then evacuated. The urethra is evaluated, then a silicon urethra catheter is inserted. The stone composition was analyzed, and Ca oxalate was found.

The wound was washed with 0.9 % normal saline in the urethra, and sutured the bladder in two layers with Vicryl 3.0. Penrose drain was placed on the perineal surgical site. The drain was placed on the cavum retzii. The next step is to install a silicone urethral catheter No. 20 fr, then suture the urethra with Vicryl 3.0 disconnected. Operation time is 75 min. The Redon drain was removed when the patient was discharged from the hospital. The patient was discharged from the hospital on the 2nd post-operative day and provided with regular wound care education at home. The catheter is removed on day 14. When the evaluation of dry wounds was obtained, the patient can have spontaneous micturition and dissolved hydronephrosis. UVJ stone was expelled when we explored the bladder stone. The patient chooses conservative for the kidney stone, with no complaints about kidney stones. There is no imaging after surgery; bladder, urethral, and UVJ stones were extracted.

## 3 Discussion

Patient management is currently carried out towards endourology, but open surgery can still be done. Additional interventions should be reviewed with the urologist immediately, and an appropriate treatment strategy should be created based on the patient's risk factors, medical history, acute presentation, and the urologist's expertise and preferences. We performed two procedures on this patient, namely vesicolithotomy and urethrotomy. Other case reports also use external basic procedures, such as urethrotomy combined with open vesicolithotomy in an 18-month-old male infant. It is a practical option in the hands of general surgeons working in first-level hospitals [13]. If the location of the urethral stone is found by palpation or KUB photo, a urethrotomy can be performed. A urethral incision can be performed directly over the stone or in a healthy urethra close to it, after which the urolith is removed [14].

The patient, in this case, gets improvement. The stone composition was analyzed, and Ca oxalate was found. Patients with a history of calcium stones should be adequately hydrated (increasing fluid intake to 2.5–3.5 l per day to minimize recurrence) and avoid oxalate-rich foods and drinks. Most acute urinary stones will come to the emergency department, which requires effective communication. Proper, rapid laboratory, urinalysis, and imaging are important interventions in patients with increased disease rates [1].

The patient has the main complaints of not being able to urinate spontaneously for a day, frequent discharge of stones from the penis, and lastly, accompanied by hematuria. Some complaints have existed for a long time, namely complaints of pus coming out from around the perineum, pus also coming out of the scrotum, and there is still blood that comes out without stopping. Another 32-year-old patient with a similar case came in with a history of lower abdominal pain and dysuria for two years. The patient has a history of urinary tract infection recurrence for the last year and hematuria [15].

The results of the bladder examination showed the presence of a giant bladder stone (1278 HU) 4.1 × 7.2 cm. Enormous bladder stones measuring >100 g are uncommon in Western medicine, and they are typically linked with bladder outlet obstruction, urinary tract infection, or intravesical foreign bodies. Previous case reports revealed one case in a 53-year-old man with a massive bladder stone measuring 600 g. He received a suprapubic cystolithotomy, had no severe surgical problems, and was discharged with markedly improved urine flow [4]. In the literature, if the size is>3 cm, it is called a large bladder stone [16]. Nugroho et al. (2019). conducted a case study on a 32-year-old man who had experienced dysuria and lower abdomen pain for the previous two years. The extracted bladder stone found weighed 832 g and measured 12.6 × 9.8 × 7.5 cm [15]. It is uncommon for bladder stones to weigh >100 g [3]. The results of the urethral examination showed the presence of a stone (HU 1275) 4.3 × 4.2 cm, without mass. The majority of urethral stones are small; however, occasionally, huge stones are found that complicate the course of treatment [17]. In this case, it is located posteriorly. Imaging studies often localize these stones, generally in the posterior urethra or anterior urethra, although sometimes computerized tomography may not identify affected urethral stones.

The patient was treated with combined vesicolithotomy and urethrotomy procedures for the management of complex cases. Bladder stones can be managed alone or in conjunction with extracorporeal or endocorporeal lithotripsy, endoscopic removal via a retrograde or antegrade strategy, and open vesicolithotomy. Endourology is the initial choice of treatment for most urinary calculi. However, open surgery remains the most recommended treatment for large and complex bladder stones [18]. On the other hand, open surgery, such as urethrotomy, is indicated for big urethral stones because, in most of those circumstances, standard minimally invasive endoscopic interventions, such as forceps or basket removal, or endoscopic push-back with lithotripsy, are not possible [19]. In our case, due to the concurrent large bladder and urethral stones, we performed open surgery, vesicolithotomy, and urethrotomy. We documented the safety of vesicolithotomy and urethrotomy in our case, in which our patient had minor postoperative problems and got only a grade 1 Clavien-Dindo classification.

## 4 Conclusion

Large stones in the bladder and urethra are rare. Current management is towards endourology, but open surgery can still be done. Management is adjusted to the results of the patient's examination.

## Ethical approval

Ethical approval has been acquired in this study by Health Research Ethics Committee of Dr. Soetomo General-Academic Hospital, Surabaya, Indonesia.

## Funding

The author(s) received no financial support for the research.

## Author contribution

Y.A.A: Conceptualization, Methodology, Investigation, Data Curation, Writing original draft, Reviewing and Editing

R.S: Conceptualization, Methodology, Investigation, Data Curation, Writing original draft, Reviewing and Editing

S.W: Writing original draft, Reviewing, Supervision, Validation

K.M.S: Conceptualization, Methodology, Investigation, Data Curation, Writing original draft

## Guarantor

Soetojo Wirjopranoto

## Additional information

All information is private for this paper.

## Conflict of interest statement

The authors declare no conflict of interest.

## Data Availability

No data was used for the research described in the article.
